# Double‐ring sign in granulocyte colony‐stimulating factor‐induced vasculitis

**DOI:** 10.1002/rcr2.976

**Published:** 2022-05-18

**Authors:** Reimi Mizushima, Ryota Kikuchi, Hiroyuki Takoi, Nao Shioiri, Kazutoshi Toriyama, Shinji Abe

**Affiliations:** ^1^ Department of Respiratory Medicine Tokyo Medical University Hospital Tokyo Japan

**Keywords:** double‐ring sign, granulocyte colony‐stimulating factor, lung cancer, Takayasu arteritis, vasculitis

## Abstract

If a double‐ring sign is found in contrast‐enhanced computed tomography, this should raise concern not only for Takayasu arteritis, but also for granulocyte colony‐stimulating factor‐induced vasculitis.

The double‐ring sign found in contrast‐enhanced computed tomography, which reflects inflammatory changes in the adventitia and oedema of the intima, is thought to be characteristic of Takayasu arteritis; however, herein, it was also observed for granulocyte colony‐stimulating factor‐induced vasculitis.

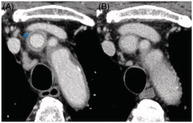

## CLINICAL IMAGE

**FIGURE 1:**
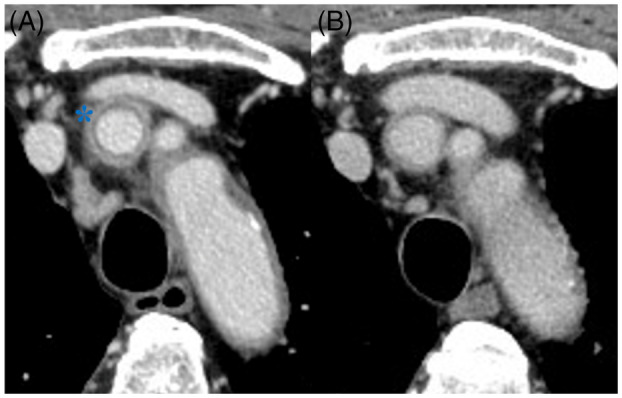
Venous phase of enhanced computed tomography (CT) findings. (A) CT performed 8 days after the administration of filgrastim revealed thickening of the brachiocephalic artery wall and enhancement with a double‐ring sign (asterisk). (B) CT performed after the administration of prednisolone demonstrated decreased arterial wall thickening without the double‐ring sign.

A 77‐year‐old male was treated with carboplatin and etoposide for a lung neuroendocrine tumour. He was administered filgrastim, a granulocyte colony‐stimulating factor (G‐CSF). Subsequently, the patient developed fever and was diagnosed with febrile neutropenia. Cefepime was administered, but the fever did not improve. Computed tomography (CT) showed thickening of the brachiocephalic artery wall with an enhancing outer ring and a poorly enhanced internal ring, described as the ‘double‐ring sign’ (Figure 1A). He had no symptoms other than fever. Human leukocyte antigens B52/62 were negative. The patient was diagnosed with filgrastim‐induced vasculitis; he did not meet the criteria for primary vasculitis, and there was no CT evidence of vasculitis before filgrastim administration. Treatment with prednisolone resolved the fever. CT showed decreased thickening of the arterial wall and was negative for the double‐ring sign (Figure 1B). G‐CSF‐induced vasculitis has been reported to occur in 0.47% of patients.^1^ The double‐ring sign, which reflects inflammatory changes in the adventitia and oedema of the intima, is thought to be characteristic of Takayasu arteritis (TA); however, herein, it was also observed for G‐CSF‐induced vasculitis.^2^ It is believed that the contrast‐enhanced outer layer reflects inflammatory changes associated with angiogenesis of the adventitia and media, and the poor contrast inner layer reflects mucin‐like and gelatin‐like oedema of the endometrium.^3^ Findings of a double‐ring sign should raise suspicion not only for TA, but also for G‐CSF‐induced vasculitis.

## AUTHOR CONTRIBUTION

Reimi Mizushima and Ryota Kikuchi designed the study. Shinji Abe, Ryota Kikuchi, Hiroyuki Takoi, Nao Shioiri and Kazutoshi Toriyama analysed the data. Reimi Mizushima and Ryota Kikuchi wrote the paper.

## CONFLICT OF INTEREST

None declared.

## ETHICS STATEMENT

The authors declare that appropriate written informed consent was obtained for the publication of this manuscript and accompanying images.

## Data Availability

The data that support the findings of this study are available on request from the corresponding author. The data are not publicly available due to privacy or ethical restrictions.
